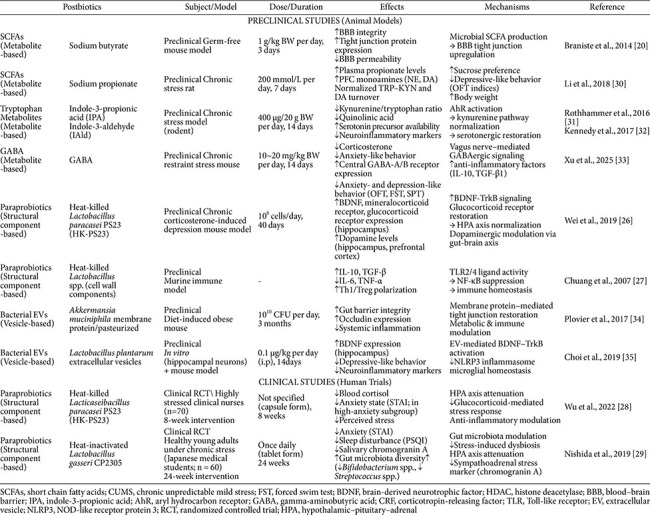# Emerging Roles of Postbiotics in Gut–Brain–Microbiome Axis Modulation and Neurobiological Pathways of Chronic Stress–Related Brain Dysfunction

**DOI:** 10.4014/jmb.2603.03010

**Published:** 2026-04-28

**Authors:** Jae Yeon Joung, Hyo Su Choi, Nam Su Oh

**Affiliations:** 1 Department of Food and Biotechnology, Korea University, Sejong 30019, Republic of Korea; 2 Institute of Natural Science, Korea University, Sejong 30019, Republic of Korea

**Keywords:** Postbiotics, Gut–Brain–Microbiome Axis, Chronic Stress, Neuroinflammation, HPA Axis, Neuroplasticity, Blood–Brain Barrier

## Abstract

Chronic psychological stress disrupts the gut-brain-microbiome axis (GBMA) through gut dysbiosis, intestinal and blood-brain barrier disruption, hypothalamic-pituitary-adrenal (HPA) axis dysregulation, and neuroinflammation, collectively impairing neurotransmitter signaling and neuroplasticity. Addressing these interconnected pathological processes requires therapeutic strategies capable of acting across multiple nodes of the GBMA simultaneously. Postbiotics, defined as preparations of inanimate microorganisms and/or their components that confer a health benefit on the host, have emerged as promising candidates for restoring GBMA homeostasis under chronic stress. Key postbiotic classes, including short-chain fatty acids, tryptophan metabolites, GABA-related compounds, heat-killed bacteria, and bacterial extracellular vesicles, attenuate neuroinflammation, reinforce barrier integrity, normalize neurotransmitter balance, and promote brain-derived neurotrophic factor (BDNF)-dependent neuroplasticity. Preclinical evidence has consistently demonstrated behavioral and neurochemical improvements following postbiotic administration, and limited clinical data suggest preliminary reductions in cortisol, inflammatory biomarkers, and stress-related symptom severity. However, clinical translation remains constrained by the absence of standardized postbiotic characterization and limited mechanistic data from human trials. This review provides an integrated account of the neurobiological pathways by which chronic stress disrupts the GBMA and examines the emerging roles of postbiotics in modulating these pathways, with the goal of informing future postbiotic-based strategies for chronic stress-related brain dysfunction.

## Introduction

Chronic psychological stress has emerged as a major global health burden, contributing to the rising prevalence of stress-related neuropsychiatric disorders including anxiety, depression, and cognitive impairment [1]. Despite decades of research, current pharmacological interventions remain limited by incomplete efficacy, adverse side effects, and high rates of treatment resistance [2], prompting increasing interest in novel therapeutic strategies targeting the biological substrates of stress vulnerability. The gut-brain-microbiome axis (GBMA) has emerged as a critical mediator of stress responses and mental health. This bidirectional communication network links the gastrointestinal tract and the central nervous system via neural, immune, endocrine, and microbial signaling pathways [3]. Chronic stress disrupts this axis by inducing gut dysbiosis, impairing intestinal barrier integrity, and triggering neuroinflammation, all of which contribute to stress-related brain dysfunction [4]. Conversely, gut microbiota-derived neuroactive metabolites, including short-chain fatty acids (SCFAs), tryptophan derivatives, and gamma-aminobutyric acid (GABA), are recognized as key regulators of neuroplasticity, neurotransmitter balance, and hypothalamic-pituitary-adrenal (HPA) axis activity [5], positioning the gut microbiota as a promising therapeutic target. While probiotics and prebiotics have shown beneficial effects on gut microbiota and stress-related outcomes, their clinical translation has been hampered by strain-specific variability, susceptibility to degradation, and safety concerns in vulnerable populations [6]. These limitations have catalyzed growing interest in postbiotics, defined by the International Scientific Association for Probiotics and Prebiotics (ISAPP) as preparations of inanimate microorganisms and/or their components that confer a health benefit on the host [7]. Postbiotics offer enhanced stability, precise dosing, and a favorable safety profile compared to live microorganism-based interventions [8]. Several reviews have recently addressed the relationship between postbiotics and the microbiota-gut-brain axis [9, 10]. However, these reviews have primarily focused on general postbiotic effects without systematically linking specific postbiotic classes to the distinct neurobiological pathways disrupted by chronic psychological stress. The present review addresses this gap by providing a mechanistically integrated account of how chronic stress impairs the GBMA through dysbiosis, barrier dysfunction, HPA axis dysregulation, neuroinflammation, and impaired neuroplasticity, and by systematically mapping how specific postbiotic classes modulate each of these disrupted pathways. Specifically, this review aims to provide an integrated account of the neurobiological pathways by which chronic stress disrupts the GBMA, and to examine the emerging roles of postbiotics in modulating these pathways, with the goal of informing future postbiotic-based strategies for stress-related brain dysfunction.

## The Gut–Brain–Microbiome Axis and Neurobiological Pathways Underlying Chronic Stress–Related Brain Dysfunction

### Gut–Brain–Microbiome Axis: Bidirectional Communication and Neuroactive Signal Production

**Fig. 1:**
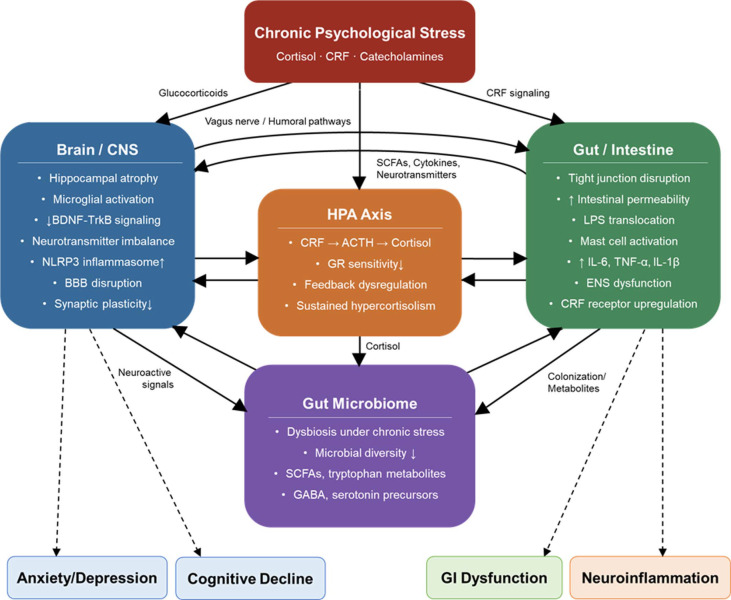
Gut–brain–microbiome axis: bidirectional communication pathways and chronic stress–induced neurobiological dysfunction. BBB, blood–brain barrier; BDNF, brain-derived neurotrophic factor; CRF, corticotropin-releasing factor; ENS, enteric nervous system; GR, glucocorticoid receptor; HPA, hypothalamic–pituitary–adrenal; LPS, lipopolysaccharide; SCFAs, short-chain fatty acids.

The gut-brain-microbiome axis (GBMA) constitutes a dynamic, bidirectional communication network integrating the central nervous system (CNS), enteric nervous system (ENS), immune system, and gut microbiota through neural, endocrine, and humoral signaling pathways, as schematically depicted in Figure 1 [3]. The vagus nerve serves as a primary afferent conduit, relaying microbial and intestinal signals directly to the brainstem, while the hypothalamic-pituitary-adrenal (HPA) axis mediates neuroendocrine feedback between the brain and the gut [11]. Humoral pathways further contribute through the systemic circulation of gut-derived metabolites, hormones, and immune mediators.

A central feature of this axis is the capacity of gut microbiota to produce neuroactive substances that influence CNS function. Short-chain fatty acids (SCFAs), including butyrate, propionate, and acetate, are generated through microbial fermentation of dietary fiber and promote epithelial integrity, modulate immune tone, and reduce neuroinflammation via epigenetic regulation and G-protein-coupled receptor pathways [12]. The tryptophan–kynurenine-serotonin metabolic pathway represents another critical interface, wherein gut bacteria regulate the availability of tryptophan for serotonin synthesis and kynurenine metabolism, thereby influencing mood, cognition, and stress reactivity [13]. Additional neuroactive metabolites, including γ-aminobutyric acid (GABA) and dopamine precursors produced by specific microbial taxa, further underscore the microbiota's capacity to modulate neurotransmitter systems. However, the production of these neuroactive metabolites is markedly disrupted under conditions of chronic psychological stress. Stress-induced alterations in gut microbiota composition reduce the availability of SCFAs and tryptophan-derived serotonin precursors, thereby impairing neurotransmitter balance and compromising the gut microbiota's capacity to support CNS homeostasis.

Chronic stress promotes gut epithelial barrier disruption through corticotropin-releasing factor (CRF) signaling and glucocorticoid exposure, which downregulate the expression of tight junction proteins such as occludin and claudin-5, thereby increasing paracellular permeability. Disruption of gut epithelial barrier integrity constitutes a pivotal mechanism linking gut microbiota dysfunction to CNS pathology. Increased intestinal permeability allows translocation of microbial products such as lipopolysaccharide (LPS) into systemic circulation, triggering TLR4-NF-κB-mediated immune activation and the release of pro-inflammatory cytokines including IL-6, TNF-α, and IL-1β [14]. These peripheral inflammatory signals access the CNS via circumventricular organs and activated immune cells, ultimately driving microglial activation and neuroinflammation.

### Chronic Stress-Induced Dysbiosis and HPA Axis Dysregulation

Chronic psychological stress profoundly disrupts gut microbiota composition through activation of the HPA axis and sympatho–adrenomedullary system. At the physiological level, sustained release of CRF acts on CRF receptors expressed in the gut to directly alter intestinal motility and mucosal secretion, increasing the rate of luminal transit and reducing mucus production, thereby destabilizing the habitat of commensal microorganisms [15]. Concurrently, sympatho-adrenomedullary activation elevates circulating catecholamines, which modulate microbial gene expression and virulence through adrenergic signaling, while also impairing mucosal blood flow and immune surveillance [15]. Glucocorticoids released during sustained HPA axis activation further suppress secretory IgA production and alter bile acid composition, collectively creating an intestinal microenvironment unfavorable to commensal bacteria. Consequently, beneficial SCFA-producing taxa such as *Faecalibacterium prausnitzii*, *Roseburia*, and *Akkermansia muciniphila* are depleted, while pathobionts including *Escherichia-Shigella* expand, collectively reducing microbial diversity and metabolic output [16]. Concurrently, tryptophan metabolite-producing taxa, including *Clostridium sporogenes*, *Lactobacillus* spp., and *Bifidobacterium* spp., which are responsible for generating indole derivatives and serotonin precursors, are similarly reduced under chronic stress conditions, further impairing serotonergic signaling and kynurenine pathway regulation [11].

This stress-induced dysbiosis initiates a self-perpetuating cycle of gut–brain dysfunction, as illustrated in Fig. 1. Reduced SCFA and tryptophan metabolite production weakens the mucosal barrier, facilitating microbial product translocation and amplifying systemic inflammation, which in turn further activates the HPA axis [4]. At the CNS level, chronic glucocorticoid exposure progressively impairs glucocorticoid receptor sensitivity in the hippocampus and prefrontal cortex, compromising negative feedback regulation of the HPA axis and resulting in sustained cortisol hypersecretion [17]. Prolonged glucocorticoid exposure also induces hippocampal neuronal atrophy and volumetric reduction, contributing to the cognitive and affective deficits characteristic of chronic stress-related disorders.

### Neuroinflammation, Neurotransmitter Disruption, and Impaired Neuroplasticity

Peripheral inflammatory signals generated through stress-induced dysbiosis and barrier dysfunction converge on the CNS to drive neuroinflammation. Circulating cytokines and LPS activate microglia, the resident immune cells of the brain, promoting a pro-inflammatory phenotype characterized by NLRP3 inflammasome activation and sustained release of IL-1β, IL-6, and TNF-α [18]. This neuroinflammatory milieu disrupts neural circuit homeostasis and has been causally linked to the pathophysiology of anxiety, depression, and cognitive impairment.

Concurrent disruption of neurotransmitter systems further aggravates stress-related brain dysfunction. Chronic stress and associated neuroinflammation shift tryptophan metabolism away from serotonin synthesis toward the kynurenine pathway, increasing the production of neurotoxic metabolites such as quinolinic acid while depleting neuroprotective kynurenic acid [13]. Serotonergic, dopaminergic, and GABAergic signaling are correspondingly impaired, undermining mood regulation, reward processing, and inhibitory neural control. Dysregulation of these systems collectively contributes to the affective and cognitive symptoms observed in stress-related neuropsychiatric disorders.

Chronic stress also exerts pronounced effects on neuroplasticity through suppression of brain-derived neurotrophic factor (BDNF) expression. Reduced BDNF–TrkB signaling impairs hippocampal neurogenesis, dendritic arborization, and synaptic plasticity, resulting in structural and functional deterioration of brain regions critical for memory consolidation, emotional regulation, and stress coping [19]. These neuroplastic deficits are closely correlated with the severity of stress-related behavioral dysfunction and represent key targets for therapeutic intervention.

### Gut Barrier and Blood–Brain Barrier Disruption

The intestinal epithelial barrier and the blood-brain barrier (BBB) share structural and functional parallels, and both are vulnerable to disruption under conditions of chronic stress and systemic inflammation. At the intestinal level, CRF signaling and glucocorticoid exposure downregulate the expression of tight junction proteins including occludin and claudin-5, increasing paracellular permeability and facilitating the translocation of luminal antigens and microbial metabolites into systemic circulation [15]. Concurrently, peripheral inflammatory mediators generated by gut barrier dysfunction compromise BBB integrity through analogous mechanisms, reducing tight junction protein expression in cerebral endothelial cells and increasing CNS exposure to neurotoxic circulating factors [20].

This parallel deterioration of gut and brain barriers creates a bidirectional amplification loop. Peripheral inflammation originating from a leaky gut exacerbates BBB permeability, while central neuroinflammation further sensitizes the gut–brain axis to subsequent stressors, sustaining and deepening neuroimmune dysregulation [14, 20]. The resulting state of concurrent barrier dysfunction across the gut-brain axis perpetuates neuroinflammation, accelerates neurotransmitter imbalance, and reinforces HPA axis hyperactivation, collectively driving the progression of chronic stress-related brain dysfunction.

## Postbiotics, Definition, Classification, and Bioactive Components

### Definition and Conceptual Framework

The ISAPP definition of postbiotics formally distinguishes them from probiotics, which require microbial viability to exert their effects, and from prebiotics, which function by selectively stimulating the growth or activity of beneficial microorganisms [21]. Because postbiotics do not rely on live bacteria, they offer several practical advantages over conventional microbiome-based interventions, including greater physicochemical stability, longer shelf life, precise and reproducible dosing, and a more favorable safety profile, particularly for immunocompromised or critically ill individuals in whom administration of live microorganisms may pose risks [8]. These characteristics make postbiotics well-suited for standardized clinical application and pharmaceutical development.

### Major Classes of Postbiotics Relevant to Neuromodulation

**Fig. 2:**
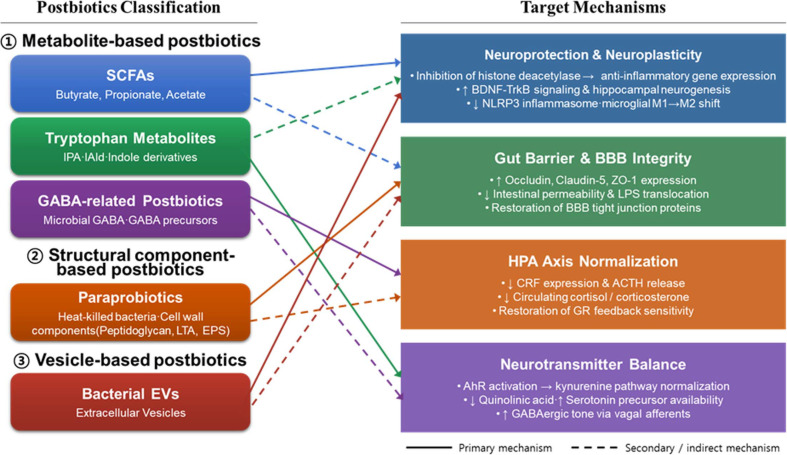
Postbiotic classes and their modulatory mechanisms on the gut–brain–microbiome axis under chronic stress. SCFAs, short-chain fatty acids; IPA, indole-3-propionic acid; IAld, indole-3-aldehyde; GABA, gamma-aminobutyric acid; LTA, lipoteichoic acid; EPS, exopolysaccharides; EVs, extracellular vesicles; BDNF, brain-derived neurotrophic factor; TrkB, tropomyosin receptor kinase B; NLRP3, NOD-like receptor protein 3; BBB, blood–brain barrier; CRF, corticotropin-releasing factor; ACTH, adrenocorticotropic hormone; GR, glucocorticoid receptor; AhR, aryl hydrocarbon receptor.

Postbiotics encompass a structurally and functionally diverse range of bioactive compounds, which can be broadly categorized into three major classes based on their origin and molecular nature (Fig. 2). The first class comprises metabolite-based postbiotics, which are soluble factors secreted or released during microbial fermentation. Among these, SCFAs, particularly butyrate, propionate, and acetate, are the most extensively studied for their neuromodulatory properties [12]. Additional metabolite-based postbiotics relevant to brain function include microbial peptides, GABA, indole derivatives, and tryptophan metabolites such as indole-3-propionic acid and indole-3-aldehyde, all of which interact with key nodes of the gut–brain–microbiome axis [22]. The second class consists of structural component-based postbiotics, derived from the microbial cell itself following inactivation. These include heat-killed or UV-inactivated whole bacteria, referred to as paraprobiotics, as well as isolated cell wall constituents such as peptidoglycan, lipoteichoic acid, and exopolysaccharides (EPS) [23]. Despite the loss of viability, these structural components retain immunomodulatory activity through pattern recognition receptors including TLRs, enabling them to regulate innate immune responses at both the intestinal and systemic levels. The third class encompasses vesicle-based postbiotics, represented by bacterial extracellular vesicles (EVs). EVs are of particular interest in the context of gut–brain communication, as they can traverse the intestinal epithelium, enter systemic circulation, and potentially reach the central nervous system, where they may exert neuroprotective and anti-inflammatory effects. The cargo of bacterial EVs includes proteins, lipids, nucleic acids such as small RNAs, and bioactive metabolites, each contributing to distinct functional outcomes. Proteinaceous and lipopolysaccharide-associated components modulate intestinal immune responses through TLR-mediated signaling, while small RNA cargo can regulate host gene expression post-transcriptionally. Of particular relevance to stress-related brain dysfunction, bacterial EVs have been shown to reinforce gut and blood–brain barrier integrity by upregulating tight junction protein expression, and to exert neuroprotective effects through activation of BDNF–TrkB signaling and suppression of NLRP3 inflammasome-mediated neuroinflammation [24, 35]. As illustrated in Fig. 2, these three structural classes give rise to five functionally distinct postbiotic candidates; SCFAs, tryptophan metabolites, GABA-related compounds, paraprobiotics, and bacterial EVs, each engaging complementary molecular targets within the GBMA to collectively restore GBMA homeostasis under conditions of chronic stress.

## Preclinical and Clinical Evidence for Postbiotic Modulation of the Gut–Brain–Microbiome Axis Under Chronic Stress

### Key Findings from Animal Studies

Preclinical studies using established chronic stress models, including chronic unpredictable mild stress (CUMS) and social defeat stress, have provided substantial evidence for the efficacy of postbiotics in mitigating stress-induced GBMA dysfunction. Across these models, diverse postbiotic interventions encompassing SCFAs, heat-killed bacteria, tryptophan metabolites, and bacterial extracellular vesicles have consistently demonstrated improvements across behavioral, neurochemical, and histological outcomes, as summarized in Table 1.

Behaviorally, postbiotic-treated animals exhibited significant reductions in immobility time in forced swim tests, increased open-arm exploration in elevated plus maze paradigms, and improved performance in spatial memory tasks, collectively indicating attenuation of depression- and anxiety-like phenotypes [25, 26]. At the neurochemical level, postbiotic administration restored hippocampal BDNF expression, normalized serotonin and corticosterone levels, and reduced pro-inflammatory cytokine concentrations in both serum and brain tissue. Histologically, improvements in intestinal tight junction protein expression, reductions in microglial activation markers, and preservation of hippocampal neuronal density were consistently observed [26, 27]. Notably, butyrate supplementation in CUMS models attenuated depressive-like behavior through histone deacetylase inhibition and restoration of BDNF-TrkB signaling, while heat-killed *Lactobacillus* preparations normalized corticosterone feedback sensitivity and suppressed hippocampal neuroinflammation in social defeat paradigms [25, 27].

Collectively, these preclinical findings support a mechanistic framework in which postbiotics act through complementary pathways to restore GBMA homeostasis disrupted by chronic stress.

### Human Clinical Trials: Current Landscape

Clinical evidence for postbiotic efficacy in stress-related brain dysfunction, while still nascent, is beginning to accumulate. Randomized controlled trials examining the effects of paraprobiotic preparations, specifically heat-killed or heat-inactivated bacterial strains, in individuals experiencing chronic psychological stress have reported promising outcomes, with key findings presented in Table 1.

Wu *et al*. [28] demonstrated that 8-week supplementation with heat-killed *Lacticaseibacillus paracasei* PS23 (HK-PS23) significantly reduced blood cortisol levels in highly stressed clinical nurses, and further improved anxiety states in the subgroup with higher baseline anxiety scores, suggesting HPA axis attenuation as a primary mechanism of action. Nishida *et al*. [29] reported that 24-week administration of heat-inactivated *Lactobacillus gasseri* CP2305 tablets in healthy young adults under chronic academic stress significantly reduced anxiety and sleep disturbance, as assessed by validated psychometric instruments, and lowered salivary chromogranin A, a sympathoadrenal stress marker. Notably, 16S rRNA gene sequencing revealed that CP2305 supplementation attenuated stress-induced gut dysbiosis, preserving *Bifidobacterium* spp. abundance and suppressing *Streptococcus* spp. expansion, suggesting that postbiotic interventions may support a more resilient microbial ecosystem even in the absence of live bacterial colonization [29].

However, the mechanistic pathways underlying these clinical observations remain incompletely defined, and the current evidence base is limited by small sample sizes and a restricted number of verified postbiotic RCTs, underscoring the need for larger, well-designed human trials.

### Gaps and Limitations in Current Evidence

Despite promising preclinical and emerging clinical findings, several critical gaps limit the current evidence base for postbiotics as therapeutic modulators of stress-related GBMA dysfunction. A primary challenge is the absence of standardized definitions and characterization criteria for postbiotic preparations across studies, resulting in substantial heterogeneity in the compounds investigated and rendering cross-study comparisons unreliable [7]. The majority of clinical trials conducted to date have been limited by small sample sizes, short intervention durations, and inadequately powered designs, reducing their capacity to detect clinically meaningful effects or establish causal relationships. Notably, the current review identified only two human RCTs involving postbiotic preparations with stress-related outcomes, which restricts the strength of clinical claims. The observations regarding reductions in cortisol, inflammatory biomarkers, and stress-related symptom severity could therefore be regarded as preliminary, and further well-designed human trials using clearly defined postbiotic interventions are needed. Furthermore, the mechanistic pathways through which specific postbiotic compounds exert their neuromodulatory effects in humans remain incompletely defined, as most mechanistic data are derived from rodent models with limited translational validity.

Future research should prioritize the development of standardized postbiotic characterization frameworks and adopt multi-omics approaches integrating metagenomics, metabolomics, and neuroimaging to elucidate the gut–brain pathways engaged by specific postbiotic classes in human populations. Large-scale, adequately powered randomized controlled trials with clearly defined postbiotic preparations, validated psychiatric outcome measures, and longitudinal follow-up are essential to establish the clinical efficacy and optimal therapeutic parameters of postbiotic interventions for stress-related brain dysfunction [7].

## Conclusion

Chronic psychological stress disrupts the GBMA through interconnected pathological processes, including dysbiosis, intestinal and blood-brain barrier disruption, HPA axis dysregulation, neuroinflammation, and impaired neuroplasticity. The present review has outlined how these mechanisms operate in concert to drive stress-related brain dysfunction, and has examined emerging evidence supporting postbiotics as interventions capable of targeting multiple nodes of this disrupted axis simultaneously.

Postbiotics, encompassing SCFAs, heat-killed bacteria, tryptophan metabolites, GABA-related compounds, and bacterial EVs, demonstrate the capacity to attenuate neuroinflammation, reinforce barrier integrity, normalize neurotransmitter signaling, and promote BDNF-dependent neuroplasticity. Their favorable safety and stability profile relative to live microorganism-based interventions further strengthens their translational potential. While preclinical evidence has consistently demonstrated behavioral and neurochemical improvements following postbiotic administration, clinical evidence remains limited by small sample sizes, heterogeneous preparations, and insufficient mechanistic data from human populations.

Addressing these gaps through standardized postbiotic characterization frameworks, multi-omics approaches, and large-scale randomized controlled trials will be essential to fully establish the therapeutic value of postbiotics for chronic stress-related brain dysfunction. As research in this area continues to grow, postbiotics may represent a promising complementary or adjunctive approach, pending further clinical validation to preventing and treating chronic stress-related brain dysfunction.

## Figures and Tables

**Table 1:** Summary of preclinical and clinical studies on postbiotic modulation of the gut–brain–microbiome axis under chronic stress.